# The Effect of Zinc, Copper, and Silver Ions on Oat (*Avena sativa* L.) Androgenesis

**DOI:** 10.3390/plants10020248

**Published:** 2021-01-28

**Authors:** Marzena Warchoł, Katarzyna Juzoń, Kinga Dziurka, Ilona Czyczyło-Mysza, Kamila Kapłoniak, Izabela Marcińska, Edyta Skrzypek

**Affiliations:** Department of Biotechnology, The *Franciszek Górski* Institute of Plant Physiology, Polish Academy of Sciences, Niezapominajek 21, 30-239 Krakow, Poland; k.juzon@ifr-pan.edu.pl (K.J.); k.dziurka@ifr-pan.edu.pl (K.D.); i.czyczylo@ifr-pan.edu.pl (I.C.-M.); k.kaploniak@ifr-pan.edu.pl (K.K.); i.marcinska@ifr-pan.edu.pl (I.M.); e.skrzypek@ifr-pan.edu.pl (E.S.)

**Keywords:** androgenesis, green plantlets, pretreatment, Zn^2+^, Cu^2+^, and Ag^+^ ions

## Abstract

Oat (*Avena sativa* L.) cultivars ‘Bingo’ and ‘Chwat’ were used to compare the embryogenesis competence of another culture. Despite the embryo-like structures obtained from both tested cultivars, only ‘Chwat’ produced green plantlets, which confirmed the cultivar dependency. ‘Chwat’ produced the highest number of embryo-like structures and green plantlets (0.7/100 anthers and 0.1/100 anthers, respectively). The embryo-like structure formation also depended on cold pretreatment combined with Cu^2+^, Zn^2+^, or Ag^+^ ion supplementation, which was applied during the tiller pretreatment or added to the induction media. The highest number of embryo-like structures (2.1/100 anthers) were observed on anthers derived from the tillers kept in a 50% Hoagland medium with the addition of 10 µM of CuSO_4_. In turn, the induction media supplemented with the ions Cu^2+^, Zn^2+^, or Ag^+^ increased neither the number of embryo-like structures nor the green plantlet production compared to the control conditions. However, such ion applications turned out to be most effective when the induction medium was enriched with 25 µM of AgNO_3_ and left to obtain the highest number of embryo-like structures and green plantlets (0.8/100 anthers and 0.2/100 anthers, respectively). Therefore, more attention should be paid to the possibilities of adjusting the media nutrient composition, as this may be the only way to significantly increase the efficiency of this method.

## 1. Introduction

Androgenesis is defined as a developmental pathway leading to obtaining a male-derived haploid embryos [1]; presently, it is widely used in the production of doubled haploid (DH) plants in commercial breeding systems. This biotechnological method constitutes an alternative to the numerous cycles of inbreeding or backcrossing required to obtain valuable pure lines used in conventional plant breeding [2]. The most common technique of androgenesis is the isolation of intact anthers, and currently the method is used in a number of crop species, including wheat (*Triticum aestivum* L.), barley (*Hordeum vulgare* L.), and rice (*Oryza sativa* L.) [3]. For oat (*Avena sativa* L.), DH line production via both androgenesis in anther and microspore culture [4,5,6] as well as distant crossing with maize [7,8,9,10] or pearl millet [11] has been used. Unfortunately, the results of the investigation conducted so far indicate that oat is recalcitrant to haploidization; moreover, the process efficiency of this species is strongly dependent on the genotype [6,12]. Sidhu et al. [7] observed frequencies of haploid production with the oat × maize technique of between 0.8% and 6.7% haploid plants per pollinated floret, while the best recoveries reported in anther culture reached up to 30 green regenerants per 100 isolated anthers [5]. Recently, Warchoł et al. [6] noted the DH production with the highest efficiency at 0.5 plants per 100 anthers for the cultivar ‘Akt’. Such a low haploid plant regeneration rate substantially limits the incorporation of this technique into breeding programs and makes it economically unfeasible. For this reason, it seems necessary to conduct further research that may contribute to increasing the efficiency of obtaining oat DH plants. Nowadays, more than 250 plant species have been regenerated using the anther culture technique, mainly from Solanaceae, Brassicaceae, and Gramineae. However, many important species—for example, woody plants or Leguminosae—still remain resistant to androgenesis induction [13,14].

Androgenesis, like other haploid inducing techniques, is influenced by several biotic and abiotic factors. The genotype, physiological state, and growth conditions of donor plants; stage of pollen development; pretreatment of flower buds or anthers; in vitro culture medium composition; and physical factors during tissue culture together with their interactions significantly affect the response of anthers in in vitro culture [15,16]. The application of suitable physiochemical factors promotes a stress response that arrests microspores or young pollen grains in their gametophytic pathway. The switch from gametophytic to sporophytic routes, named the “development window” by Smykal and Pechan [17], is relatively limited and happens only between the mononucleate and median binucleate stages of microspores [18]. However, modifications of the stress pretreatment [4,19], carbohydrate source [20,21], or growth regulators of culture media [6,22,23] have led to a significant efficiency improvement of both the androgenesis induction and regeneration of haploid plants (HP). Although in most studies little attention has been paid to the micronutrient composition of the media, Kaushal et al. [24] emphasized that the content of macro- or microelements in the induction medium not only provides nutrition to the microspores or directs the pathways of embryo development but may also determine whether or not the androgenesis will be initiated. Among them, zinc and copper have been indicated as more essential for plant growth and development, are included in all tissue culture media, and are also used in cereal androgenesis [25,26]. Copper in the culture medium increases the yield of plant regeneration from callus cultures [27] and allows the production of green plantlets [28]. In cereals, copper exhibits a key role during pollen development [29]. Together with zinc, it is involved in many physiological processes, especially in chlorophyll synthesis and photosynthesis [30,31]. Moreover, Zn^2+^ plays a fundamental role in biochemical cellular processes, such as auxin metabolism and response to oxidative stress [32]. The tissue culture media are usually supplied with zinc sulfate (ZnSO_4_) and copper sulfate (CuSO_4_) in concentrations of 0.1–8.6 mgL^−1^ and 0.03–0.2 mgL^−1^, respectively [33]. However, as heavy metals at high concentrations, these ions might be toxic and mutagenic [34,35]. Demands for these micronutrients seem to be species-dependent, and the optimization of their concentration in the media has led to the improvement of different culture systems. Unfortunately, studies on their influence on calluses, embryo-like structures (ELS), or haploid plant (HP) production in cereals are still very limited. Zn^2+^ and Cu^2+^ ions supplemented into the pretreatment solution or induction medium were examined in barley by Wojnarowiez et al. [36], Echavarri et al. [30], and Makowska et al. [37]. A concentration of Zn^2+^ from 52 to 520 μM in the culture medium resulted in the increased growth of rice [38], whereas 10 mM became toxic for tobacco [32]. Another micronutrient used in plant tissue cultures is silver nitrate (AgNO_3_), a very potent inhibitor of ethylene action. Easy water solubility and chemical stability make it very useful in the regulation of morphogenesis in vivo and in vitro [39]. AgNO_3_ has been shown to be effective in improving somatic embryogenesis and plant regeneration in a number of dicotyledonous species—for example, *Punica granatum* and *Solanum tuberosum* [40,41]. It has also been used successfully in cereal crops, such as rice [42], maize [43], pearl millet [44], and barley [45]. Recently, Shahvali-Kohshour et al. [46] reported the positive effects of silver nitrate on the anther culture of strawberry (*Fragaria × ananassa* Duch.).

Therefore, to investigate the effect of CuSO_4_, ZnSO_4_, and AgNO_3_ on the efficiency of androgenesis induction and green plantlet (GP) regeneration, two oat cultivars—‘Chwat’ and ‘Bingo’—were studied. We analyzed the influence of metal ions (i) applied during tiller pretreatment and (ii) supplemented into induction media. According to our knowledge, this is the first report where the impact of Zn^2+^, Cu^2+^, and Ag^+^ ions on oat androgenesis has been investigated.

## 2. Results

Tiller pretreatments and induction media compositions were tested in the context of the production of calluses, ELS, and GPs using the anther culture of two oat cultivars—‘Bingo’ and ‘Chwat’. The analysis of variance presented in Table 1 shows that callus production depended significantly on the pretreatment and induction medium, while ELS development depended on the cultivar and pretreatment. The number of regenerated GPs was statistically dependent on the tested cultivar.

**Figure 1:**
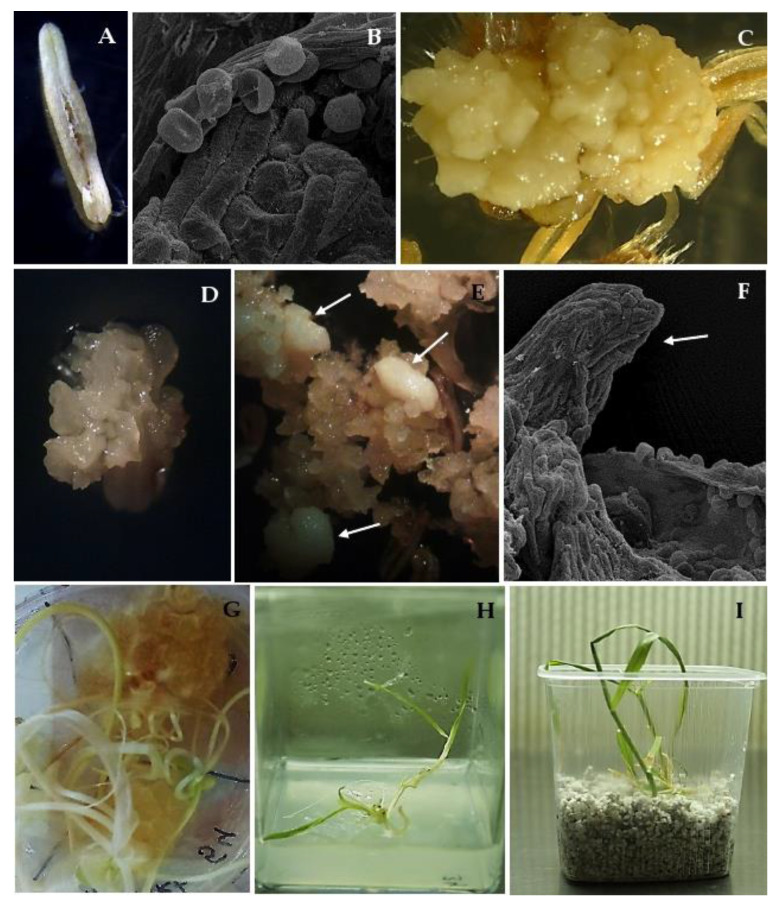
Androgenesis in oat (*Avena sativa* L.) anther culture: (**A**) dehisced anther on W14 medium; (**B**) anther with released microspores; (**C**) anthers with yellow compact calluses; (**D**,**E**) anthers with white, friable calluses with embryo-like structures indicated by arrows; (**F**) developed embryo-like structure indicated by arrow; (**G**) regenerated green plantlets on W14 medium with 25 µM of AgNO_3_; (**H**) green plantlets on MS medium; (**I**) green plantlets in perlite.

Almost nine thousand anthers from 136 panicles were isolated on the induction medium (Table 2). An almost 2.5-fold higher number of calluses were obtained in cv. ‘Chwat’ compared to cv. ‘Bingo’. The first visible observation was an enlargement in the anther size within the first two weeks after isolation (Figure 1A) which preceded the production of calluses in the following weeks of culture. Morphological observations allowed for the extraction of two types of callus: yellow compact, embryogenic (Figure 1B), and white friable, non-embryogenic (Figure 1C). Four weeks of culturing led to ELS formation (Figure 1D). In total, 38 ELS were obtained in both of the tested oat cultivars (Table 2). The highest number of ELS were obtained from cv. ‘Chwat’ (0.7 ELS/100 anthers) (Figure 1E,F), and only this cultivar produced green plantlets (0.1 GPs/100 anthers) (Table 1, Figure 1G). Despite all the developed plantlets being green and healthy (Figure 1H,I), they did not survive the colchicine treatment.

**Figure 2:**
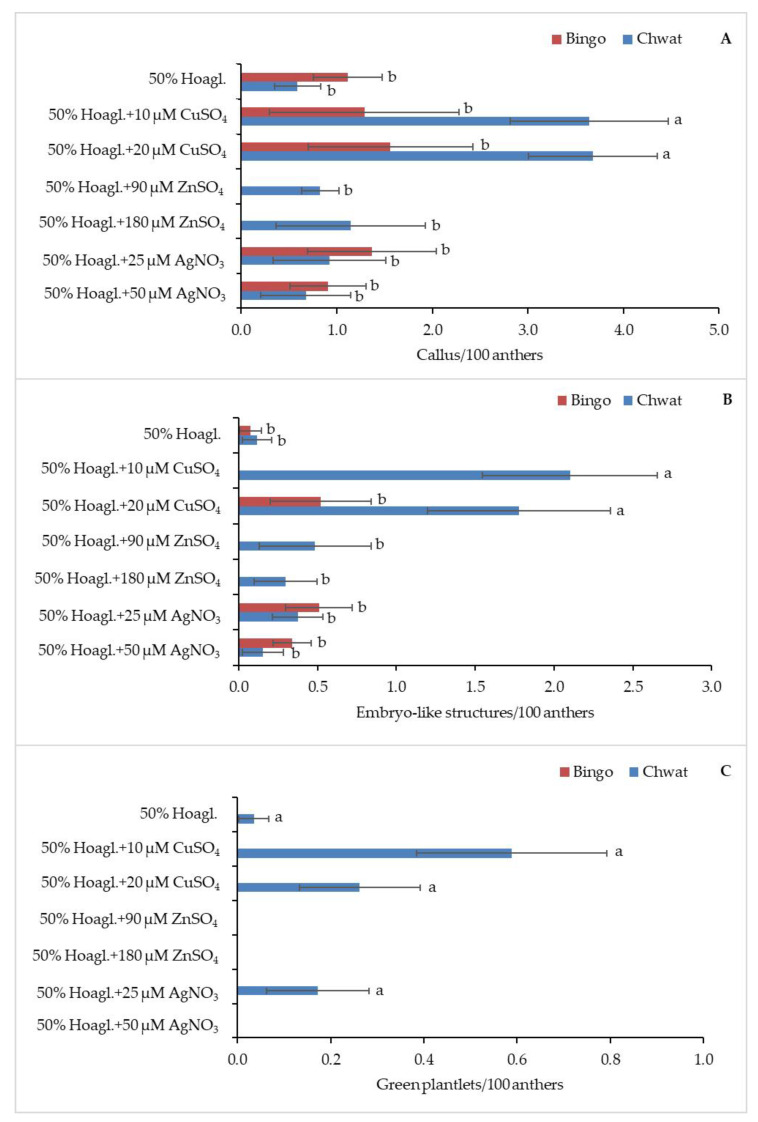
The regeneration response of oat (*Avena sativa* L.) depending on pretreatment: (**A**) number of calluses, (**B**) embryo-like structures, and (**C**) green plantlets calculated for 100 anthers. Bars represent mean values ± standard error. Significant differences between treatments at *p* < 0.05 were marked with different letters.

Five of the seven tested pretreatments caused the callogenesis of anthers in both cultivars (Figure 2A). The highest rate of callus production was observed when the panicles of cv. ‘Chwat’ were kept in 50% Hoagl.+20 µM CuSO_4_ or 50% Hoagl.+10 µM CuSO_4_ (3.7/100 anthers and 3.6/100 anthers, respectively). The anthers of cv. ‘Bingo’ did not form calluses when ZnSO_4_ was added to a 50% Hoagland medium, regardless of the applied concentration. Similar to callogenesis, the addition of copper ions to the Hoagland medium influenced the effectiveness of androgenesis (Figure 2B). The highest number of ELS were produced when the panicles of cv. ‘Chwat’ were treated with 50% Hoagl.+10 µM CuSO_4_ or 50% Hoagl.+20 µM CuSO_4_ (2.1/100 anthers and 1.8/100 anthers, respectively). Oat cv. ‘Bingo’ did not form ELS when 10 µM of CuSO_4_, or 90 or 180 µM of ZnSO_4_ were added to 50% Hoagland medium. Despite the number of GPs not being statistically significantly differentiated and dependent on pretreatment, the majority of GPs formed (0.6%) after using 50% Hoagl.+10 µM CuSO_4_ (Figure 2C).

**Figure 3:**
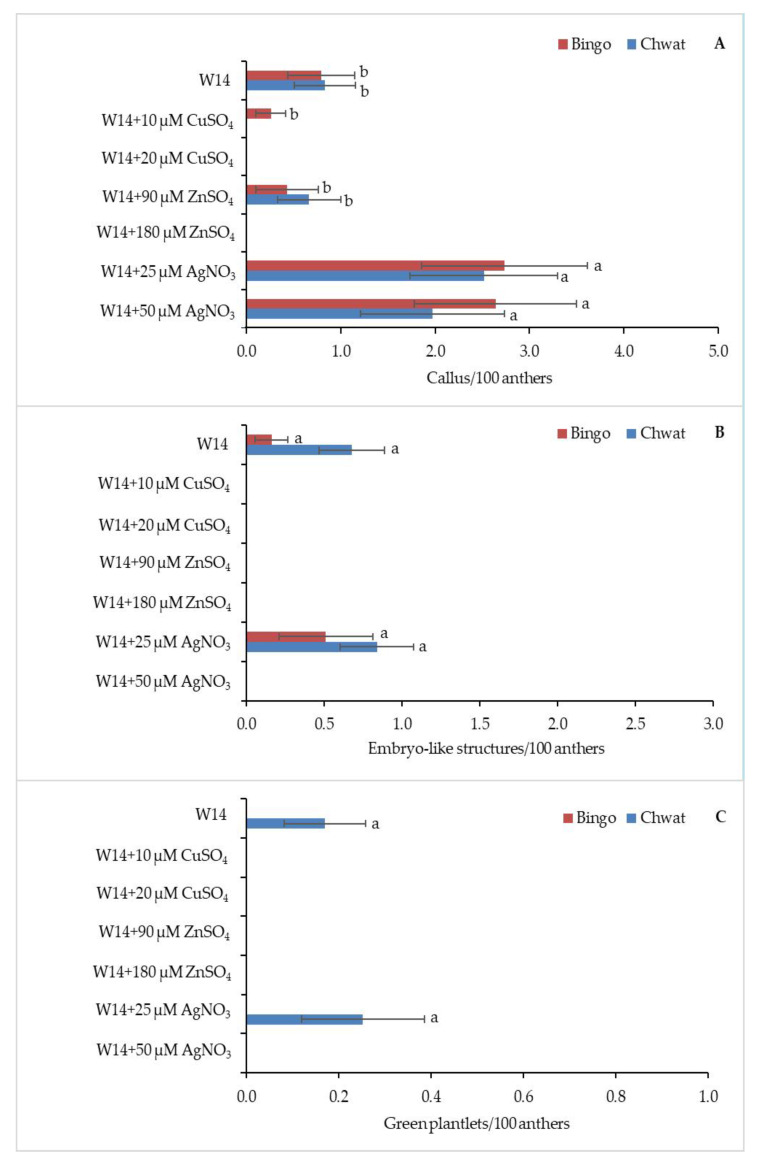
The regeneration response of oat (*Avena sativa* L.) depending on induction medium: (**A**) number of calluses, (**B**) embryo-like structures, and (**C**) green plantlets calculated for 100 anthers. Bars represent mean values ± standard error. Significant differences between treatments at *p* < 0.05 are marked with different letters.

The effect of the induction medium on androgenesis efficiency is shown in Figure 3. The anthers produced calluses on five tested media. The addition of AgNO_3_ to the induction medium led to obtaining the highest percentage of callogenesis in both tested cultivars; however, the highest efficiency was observed for cv. ‘Bingo’ on both of the tested concentrations (2.7/100 anthers and 2.6/100 anthers, respectively) (Figure 3A). ELS formation was observed only on two tested media: W14, and W14 supplemented with 25 µM of AgNO_3_. 

Although no statistically significant differences between the used induction media were observed, W14 medium supplemented with 25 µM of AgNO_3_ was more appropriate for androgenesis induction in both cultivars (Figure 3B).

Oat cv. ‘Chwat’ developed 0.8 ELS/100 anthers, while cv. ‘Bingo’ produced 0.5 ELS/100 anthers. GP formation was observed on two tested media (W14 and W14+25 µM AgNO_3_) from ELS produced by cv. ‘Chwat’ (Figure 3C).

## 3. Discussion

The production of doubled haploids through anther culture forces breeders to improve and stabilize the existing parental line with desired traits in a single year [24]. Seguí-Simarro [1] confirmed that, besides the genotype and the developmental stage of the microspore, the critical factor is the culture conditions, and, in particular, the physical and/or chemical treatment necessary to trigger microspore embryogenesis. Among external factors that can increase the frequency of haploid production, supplementation of the pretreatment solution or induction medium with micronutrients (copper, zinc, and silver) is also postulated. However, there are no reports on the effect of the above-mentioned ions on the androgenic response in oat anther culture; there are a few reports concerning these metal ions on androgenesis of *Hordeum vulgare* L. [28,30,36,37], *Oryza sativa* L. [42,47], *Triticum turgidum* [48], or *Fragaria* × *ananassa* Duch. [46]. It is well documented that in barley anther culture additional Cu^2+^ supplementation improves the effectiveness of androgenesis. A beneficial effect of copper on barley anther culture was reported by Wojnarowiez et al. [36]. The addition of copper sulfate at 10 μM during anther pretreatment and culture increased the anther response by 15% and the number of green plants by 400%. Regarding androgenesis in oat, our experiment showed that both used concentrations of copper sulfate (10 and 20 μM) added to the medium during pretreatment enhanced callogenesis, as well as ELS and GP development. These results are in accordance with studies in barley anther culture by Jacquard et al. [28]. Typical induction media for androgenesis contain low concentrations of copper, whereas the results of Dahleen [27] showed that its concentration can be up to 100 times higher, and even up to 500 times higher, in recalcitrant barley lines than the one commonly used in culture media. Similar to Makowska et al.’s [37] study in barley culture, we observed a large discrepancy in androgenic reactions to increased Cu^2+^ amounts depending on the genotype. In oat anther culture, supplementation of this ion during pretreatment only enhanced GP regeneration for cv. ‘Chwat’. These observations again confirm the significant effect of the genotype of donor plants on the androgenic response [5].

Our study shows that the addition of 90 or 180 μM ZnSO_4_ in the pretreatment medium did not increase the efficiency of the number of calluses and ELS formation in oat anther culture. The study of Echavarri et al. [30], in turn, showed that the application of Zn^2+^ ranged from 90 to 180 μM in the stress pretreatment, and induction medium significantly increased the number of embryos and green plants, in both anther and isolated microspore cultures. Zinc sulfate used in pretreatment seems to be more beneficial for the cv. ‘Chwat’, compared to the cv. ‘Bingo’. These results could indicate a higher sensitivity of ‘Bingo’ to this micronutrient, regardless of the concentration. Moreover, enriching the induction medium with a 90 µM of ZnSO_4_ already had a beneficial impact on the callus production for both genotypes. In the above-cited works, the authors emphasize the beneficial effect of both Zn^2+^ and Cu^2+^ [30,36], which was exerted mainly in barley at the initial culture phase during pretreatment and anther/microspore culture. Thus, these findings confirm that the switch of the microspore developmental program from the gametophytic to the sporophytic pathway giving rise to haploid embryos occurs in the earliest stages of androgenesis [49].

In contrast to zinc, in the present study, the addition of AgNO_3_ to the induction medium had an advantageous impact on oat anther response. Compared to the control, the enhanced callus production was obtained after supplementation with AgNO_3_ at both of the tested concentrations. The higher frequency of callus induction, observed in both cultivars, suggests that the addition of silver nitrate inhibits the ethylene production, acting as a promoter of regeneration processes in oat anthers. However, the concentration of 25 μM initiated a better response in relation to ELS formation and GP regeneration. In a previous study on white cabbage, the successful induction of regenerative processes was observed after the application of 50 μM AgNO_3_, demonstrating a high frequency (58.8%) of callus formation [50]. Likewise, Wu et al. [51] observed that supplementing culture media with silver nitrate stimulates the embryogenesis of immature wheat embryos, but only up to a concentration of 60 µM, after which it was toxic and lowered embryogenic calli formation. On the contrary, Orłowska and Bednarek [52] reported that barley plant regeneration occurs under the highest AgNO_3_ (60 µM) concentration used in this experiment. Our results support the theory of Cristea et al. [50], which suggested that the increase in anther regeneration of cabbage genotypes cultivated on media supplemented with Ag^+^ ions is due to the intervention of silver nitrate as a suppressor of ethylene. Furthermore, in our study AgNO_3_ promoted yellow compact callus induction, which was not observed in the case of the white friable structure. Yellow compact calluses were embryogenic, producing ELS and embryos, while white friable calluses did not regenerate. The present results are in agreement with earlier studies by Morris and DeMacon [53] stating that the friable wheat callus is of poor quality with a low potential for regeneration. It is considered that poor callus quality might be one of the reasons behind the poor haploid plant regeneration. Therefore, media modifications should target the production of embryogenic calluses with a good regeneration ability rather than simply inducing prolific calluses from which regeneration would not be possible [54].

In conclusion, our results demonstrate that the adjustment of copper, zinc, and silver concentrations in media may be a key point in promoting androgenesis in oat, especially when CuSO_4_ is added to the pretreatment medium or when AgNO_3_ is added to the induction medium. As oat androgenesis is still strongly cultivar-dependent, adjusting media components and their concentrations seems to be the only way to significantly improve the efficiency of this method.

## 4. Materials and Methods

### 4.1. Donor Plant Growth

Oat (*Avena sativa* L.) cultivars ‘Bingo’ and ‘Chwat’ were used as sources of anthers for studying androgenic abilities. Seeds of each genotype were sown singly into a mixture of soil and sand (3:1 (v/v) in 3 L pots. Twenty donor plants of each cultivar were grown under controlled conditions at 21/17 °C day/night with a 16 h photoperiod, in a greenhouse under natural (solar) light during the day and sodium lamps between 6 and 8 a.m. and additionally between 6 and 10 p.m. on cloudy days. Plants were fertilized with a liquid Hoagland and Arnon [55] medium once a week.

### 4.2. Pretreatment, Isolation, and Incubation

Tillers of *cv*. ‘Bingo’ and ‘Chwat’ were cut when the panicle was inside the leaf sheath and the distance from the base of the flag leaf to the penultimate leaf was a maximum of 4.0 cm [6]. Oat tillers were covered with aluminum bags and pretreated in Hoagland and Arnon [55] liquid medium alone (control) and with the addition of Cu^2+^, Zn^2+^, or Ag^+^ ions for 2 weeks at 4 °C, and then kept at 32 °C for 24 h. Next, the panicles were disinfected in 70% (v/v) ethanol (1 min), then in a 2.5% (w/v) solution of calcium hypochlorite (65% Ca(OCl)_2_ (7 min), and triple washed with sterile water [6]. For ELS induction, anthers were aseptically isolated on W14 medium [25] with 2.0 mgL^−1^ 2,4-dichlorophenoxyacetic acid (2,4-D), 0.5 mgL^−1^ kinetin, 9% maltose, and 0.6% agar (control), with the addition of Cu^2+^, Zn^2+^, or Ag^+^ ions. The media pH was adjusted to 6.0 before autoclaving (120 °C, 20 min). Cu^2+^, Zn^2+^, or Ag^+^ ions were added to the media in salt form in two concentrations: CuSO_4_ × 5 H_2_O (10 or 20 µM), ZnSO_4_ × 7 H_2_O (90 or 180 µM) and AgNO_3_ (25 or 50 µM). The arrangement of the experiment is presented in Table 3. All the salt stock solutions were filter-sterilized and added to the autoclaved medium at about 50 °C. Anther cultures were incubated in the dark at 28 ± 1 °C. Embryogenic structures were observed under a light microscope (SMZ 1500, Nikon, Tokyo, Japan) and the efficiency of androgenesis was determined as the number of calluses, ELS, and GPs obtained from 100 anthers.

### 4.3. Regeneration and Doubling the Number of Chromosomes

Developed GPs were transferred into Magenta boxes containing solidified (0.6% agar) MS medium [56] with half the original concentrations, without growth regulators. They were maintained at 21 ± 2 °C and a light intensity of 60 µmol m^−2^ s^−1^ (16/8 h light/dark). Subsequently, GPs were acclimated to ex vitro conditions by transferring them into boxes with moist perlite and then to the soil. The chromosome doubling procedure was carried out according to Warchoł et al. [10].

### 4.4. Statistical Analysis

The significance of differences in mean values was analyzed using a two-way analysis of variance (ANOVA).

The experiment was performed with the completely randomized design model application for the analysis of variance. The Duncan’s multiple range test implemented in the statistical package STATISTICA 10.0 (Stat-Soft, Inc., Tulsa OK Oklahoma, USA) was applied to detect significant differences among cultivars, pretreatment, and induction media. Significant differences between treatments at *p* < 0.05 were marked with different letters.

## Figures and Tables

**Table 1:** Two-way analysis of variance on the effect of cultivar, pretreatment, and induction medium composition on the number of calluses, embryo-like structures (ELS), and green plantlets (GPs) calculated for 100 anthers.

Dependent Variable	Independent Variable	SS	df	*F*	*p*
	Cultivar	5.610	1	3.773	0.225 ^ns^
Calluses	Pretreatment	71.118	6	3.477	0.003 **
	Induction medium	55.242	6	2.356	0.034 *
ELS	Cultivar	5.184	1	5.667	0.018 *
	Pretreatment	18.542	6	3.654	0.002 **
	Induction medium	5.388	6	0.946	0.464 ^ns^
GPs	Cultivar	0.407	1	5.031	0.026 *
	Pretreatment	0.971	6	2.031	0.065 ^ns^
	Induction medium	0.158	6	0.306	0.932 ^ns^

SS—sum of squares;df—degrees of freedom; *F*—F-test; *p*—probability of significance; ns—not significant; * significant at *p* ≤ 0.05; ** significant at *p* ≤ 0.01.

**Table 2:** The effect of the oat genotype on the efficiency of calluses, embryo-like structures (ELS), and green plantlet (GP) production in anther culture. The data show the mean values ± standard error.

Cultivar	No. of Panicles	No. of Anthers	No. of Calluses	Calluses/100 Anthers	No. of ELS	ELS/100 Anthers	No. of GPs	GPs/100 Anthers
‘Bingo’	71	4122	29	0.7 ± 0.24	5	0.1 ± 0.06	0	0
‘Chwat’	65	4779	70	1.5 ± 0.25	33	0.7± 0.10	7	0.1 ± 0.09

**Table 3:** The arrangement of the experiment.

Tillers Pretreatment 2 Weeks at 4 °C and 24 h at 32 °C	Incubation of Anther Cultures at 28 °C in Darkness
50% Hoagland’s liquid medium	W14 medium with addition of 2,4-D (2.0 mgL^−1^), kinetin (0.5 mgL^−1^) and: 10 µM CuSO_4_ × 5 H_2_O 20 µM CuSO_4_ × 5 H_2_O 90 µM ZnSO_4_ × 7 H_2_O 180 µM ZnSO_4_ × 7 H_2_O 25 µM AgNO_3_ 50 µM AgNO_3_
50% Hoagland’s liquid medium with addition of: 10 µM CuSO_4_ × 5 H_2_O 20 µM CuSO_4_ × 5 H_2_O 90 µM ZnSO_4_ × 7 H_2_O 180 µM ZnSO_4_ × 7 H_2_O 25 µM AgNO_3_ 50 µM AgNO_3_	W14 medium with addition of 2,4-D (2.0 mgL^−1^) and kinetin (0.5 mgL^−1^)

## Data Availability

All data is contained within the article. The datasets used and analyzed during the current study are available from the corresponding author on reasonable request.
